# Landscape determinants of fine-scale genetic structure of a small rodent in a heterogeneous landscape (Hluhluwe-iMfolozi Park, South Africa)

**DOI:** 10.1038/srep29168

**Published:** 2016-07-13

**Authors:** Isa-Rita M. Russo, Catherine L. Sole, Mario Barbato, Ullrich von Bramann, Michael W. Bruford

**Affiliations:** 1 Cardiff School of Biosciences, Sir Martin Evans Building, Cardiff University, Museum Avenue, Cardiff, CF10 3AX United Kingdom; 2 Department of Zoology and Entomology, University of Pretoria, Private Bag X20, Hatfield, 0028 South Africa; 3 Technische Universität Braunschweig, Institut für Geosysteme und Bioindikation, Langer Kamp 19c, Braunschweig, 38106 Germany

## Abstract

Small mammals provide ecosystem services, acting, for example, as pollinators and seed dispersers. In addition, they are also disease reservoirs that can be detrimental to human health and they can also act as crop pests. Knowledge of their dispersal preferences is therefore useful for population management and landscape planning. Genetic data were used alongside landscape data to examine the influence of the landscape on the demographic connectedness of the Natal multimammate mouse (*Mastomys natalensis*) and to identify landscape characteristics that influence the genetic structure of this species across a spatially and temporally varying environment. The most significant landscape features shaping gene flow were aspect, vegetation cover, topographic complexity (TC) and rivers, with western facing slopes, topographic complexity and rivers restricting gene flow. In general, thicket vegetation was correlated with increased gene flow. Identifying features of the landscape that facilitate movement/dispersal in *M. natalensis* potentially has application for other small mammals in similar ecosystems. As the primary reservoir host of the zoonotic Lassa virus, a landscape genetics approach may have applications in determining areas of high disease risk to humans. Identifying these landscape features may also be important in crop management due to damage by rodent pests.

Small mammals provide important ecosystem services^1^, and knowledge of their dispersal preferences is useful for population management and landscape planning^2^. Small mammals can regulate insect populations, act as pollinators and seed dispersers, support forest regeneration, aerate soil and provide food for carnivores and humans^1^. In addition, the transport of minerals from deeper layers of the soil to the surface can act as a source of nutrients for plants, and the burrowing activity of some rodent species influences surface water runoff^1^. Small mammals are also disease reservoirs that can be detrimental to human health and they can also act as crop pests^3^,^4^. Ecosystem services, food security and health risks posed by small mammals can only be regulated by managing habitats such that opportunities for migration are available, and this remains a key goal in population management^5^. It is for these reasons that it is important to understand how the landscape affects dispersal in small mammals and their associated diseases.

Dispersal in rodents is thought to be a response to a number of factors, including inbreeding avoidance, mate searching, variation in habitat quality and local competition^6^. However, despite its importance, information regarding dispersal patterns for many species, especially for small mammals, is often limited^7^.

The majority of small mammals are found in semi-isolated populations due to habitat heterogeneity^8^. In cases where the landscape between habitat patches severely restricts dispersal, patterns of population genetic differentiation should reflect landscape permeability^9^. A comparative analysis of arvicoline species (lemmings, voles and muskrats) has demonstrated frequent short distance dispersal events of hundreds of meters^6^, however, some small mammal species are capable of dispersing over much longer distances of kilometres^7^.

More specifically, the Natal multimammate mouse (Rodentia: Muridae; *Mastomys natalensis*) is an opportunistic generalist, nocturnal and omnivorous. They are to some extent dependent on water but occur in areas where water is only seasonally available^10^. “Swimming behaviour” has also been described for this species^11^. The species is a major pest in sub-Saharan Africa^3^ with a wide habitat tolerance^10^. These mice are almost ubiquitously distributed across the continent and when found in close contact with humans they have been linked to the transmission of disease, most significantly Lassa fever virus^12^,^13^, and with loss and contamination of food sources^3^. Dispersal rates and dispersal distances per generation in *M. natalensis* have been shown to be relatively high^3^ with individuals moving over distances larger than 400 m and occupying home ranges of >1 000 square meters^14^.

Previous studies on small mammals^15^ have called for additional approaches to population genetics, for example landscape genetics, to better understand dispersal in small mammal populations. Knowledge of how landscape features interact with genetic variation at both the population and individual level is required to understand gene flow and adaptation to the environment^16^. Landscape features can both restrict and facilitate the movement of individuals in natural populations^17^. Therefore, studying patterns of gene flow in relation to environmental characteristics provide indirect information on life history traits and ecological function. In addition, this provides critical information for both small mammal ecosystem services (pollinators and seed dispersers) and management^18^,^19^.

To date, small mammals have received less attention in landscape genetic studies and these studies have largely been carried out in temperate and/or developed regions, where anthropogenic effects on the landscape can be profound^20^,^21^. However, barrier effects can be just as relevant in less modified landscapes and such studies can provide relevant information for managing ecologically similar species^22^. Landscape modification can impact on the movement of species differently and it is therefore crucial to understand similarities among species for landscape planning^23^.

In this context, we investigated genetic structure and landscape heterogeneity in the Natal multimammate mouse from the Hluhluwe-iMfolozi Park (HiP), South Africa. Population structuring and division is expected to be evident in small mammals such as *M. natalensis* at small spatial scales^15^,^24^, but here we aimed to test how landscape and/or habitat features interact with dispersal. We tested the hypothesis that local-scale habitat heterogeneity (for example temperature, food availability and shelter from predators) influences patterns of genetic structure. Genetic data were therefore used alongside high-resolution landscape data to examine the influence of the landscape on the demographic connectedness of *M. natalensis* and to identify landscape characteristics that influence the genetic structure of this species across a spatially and temporally varying environment.

## Results

### Genetic results

A null allele was detected for locus MH60 and we therefore excluded this locus from all further analyses. No consistent departures from Hardy-Weinberg equilibrium were detected and no consistent linkage disequilibrium was observed. The number of alleles per locus ranged from 4 to 39 and genetic clusters showed significant differentiation (*F*_ST_ = 0.025–0.124). Allelic richness (254 diploid individuals) ranged from 3.99 to 38.98. Expected heterozygosity for the five loci in common with Brouat *et al*.^15^ was also higher in the present study. The mean expected heterozygosity varied from 0.167–0.763 for these five loci.

**Figure 1:**
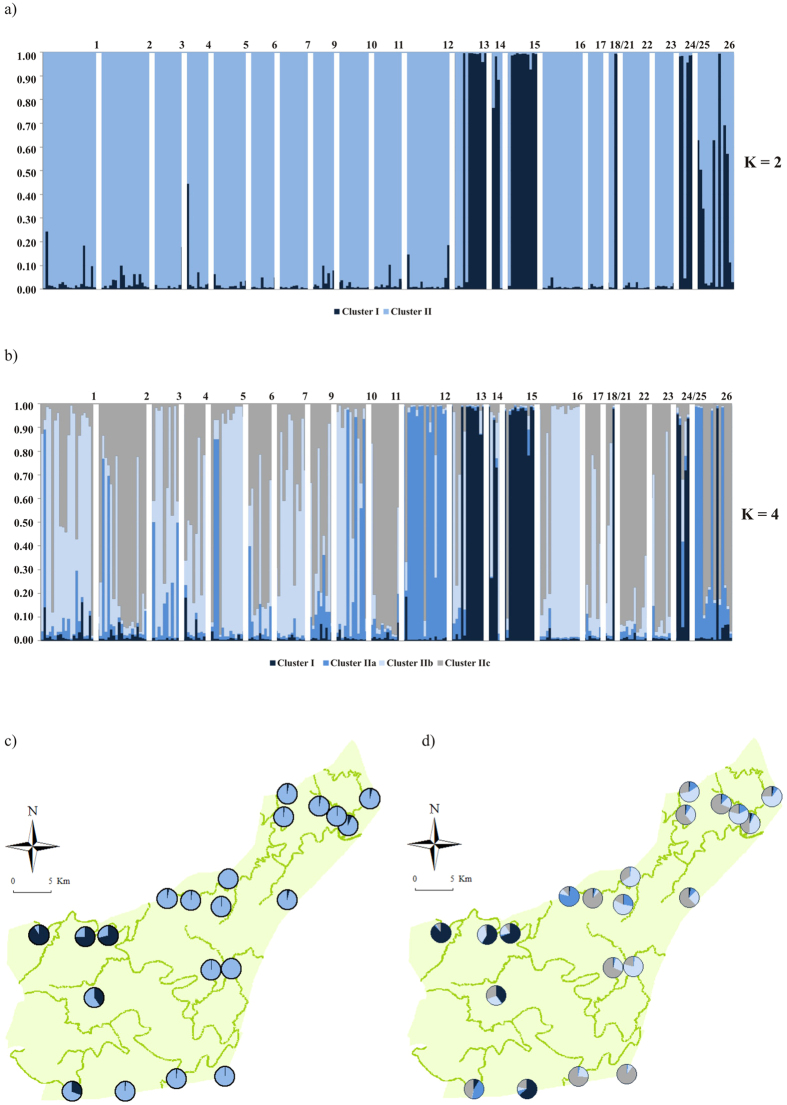
Bayesian clustering analysis in STRUCTURE. (**a**) Plot showing the individual membership coefficients for *K* = 2 when considering all rodents. Cluster I = most of the individuals south of the Black iMfolozi River, Cluster II = remainder of the individuals. (**b**) Plot of the individual membership coefficients for *K* = 4. Here, Cluster II was further divided into three clusters. Cluster IIa occurred mostly in grid 10 and 12, Cluster IIb was more common north of the White iMfolozi River and Cluster IIc was mostly distributed within the southern part of the park. See maps c (*K* = 2) and d (*K* = 4) for the geographic distribution of clusters. The colours indicate different clusters and the size of the pie charts represent the frequency of occurrence for each grid sampled. Numbers (above graphs) show sampling grid numbers as indicated in Table S1. The green lines show the four major rivers in the park. Graphs were generated in Microsoft Excel for Mac v 15.19.1 (2016) and maps were generated in ArcGIS v 10.1 (http://www.esri.com/software/arcgis/arcgis-for-desktop).

STRUCTURE analysis indicated that the most likely value of LnPr(*X*|*K*) was *K* = 7 however, the method of Evanno *et al*.^25^ indicated that the most probable number of clusters of individuals was two, followed by four (see Supplementary Fig. S4). Over all runs the probability of the data increased from *K* = 1 to *K* = 10 with a clear tendency to asymptote (see Supplementary Fig. S4). Clustering based on the Evanno method for *K* = 2 is shown in Fig. 1a,c. Cluster I predominantly originated from sites located south of the Black iMfolozi River and showed less evidence of admixture, whereas Cluster II showed scattered assignment across the area (Fig. 1a,c).

For the next highest ∆*K* value (at *K* = 4; see Supplementary Fig. S4) results showed no clear evidence of geographic structure among sites. Again, Cluster I predominantly originated from sites south of the Black iMfolozi River (Fig. 1b,d), whereas further sub-structuring of Cluster II into three additional clusters was evident (Fig. 1b,d). Cluster IIa occurred mostly in grid 10 and 12. Individuals with an admixture coefficient proportion equal or greater than 0.7 for Cluster IIb were more commonly found north of the White iMfolozi River (**χ**^2^ = 16.27, *P* < 0.001). In contrast, Cluster IIc was mostly distributed in the south of the park (**χ**^2^ = 15.43, *P* < 0.001). Genetic clusters were scattered across the park and therefore no strict geographic assortment was evident (Fig. 1d). These groups were also observed after the very low number of related individuals was removed from the analysis (not shown). When a full progressive partitioning method was implemented, the number of population clusters was *K* = 7 and in agreement with the LnPr(*X*|*K*) method, although not geographically or biologically meaningful (not shown).

### Spatial autocorrelation

**Figure 2:**
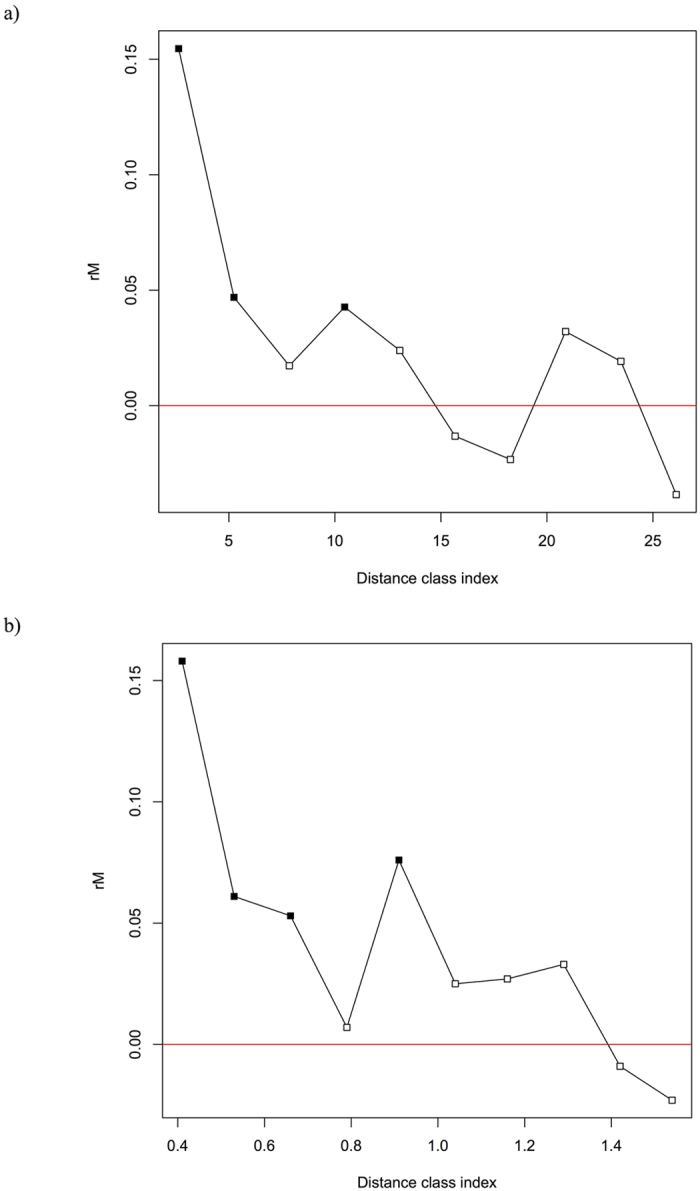
(**a**) A Mantel correlogram showing a positive correlation between genetic distance (proportion of shared alleles) and Euclidean distance in the first (0.026–2.63 km), second (2.63–5.24 km), third (5.24–7.85 km) and fourth (7.85–10.46 km) distance classes and (**b**) a Mantel correlogram between genetic distance and resistance distance showing significant and positive autocorrelation values for the smaller resistance classes. White and black squares represent non-significant and significant relationships between genetic and Euclidean/resistance distances for the different distance classes, respectively.

After adjusting the *P*-value for multiple comparisons (*P*-value = 0.017) a Mantel correlogram showed significant and positive autocorrelation values in the first (0.026–2.63 km), second (2.63–5.24 km), third (5.24–7.85 km) and forth (7.85–10.46 km) distance classes, exhibiting short distance structuring of genetic variability (Fig. 2a). Results showed a higher and significant genetic correlation among individuals than expected within some distance classes. When testing for association between genetic and resistance distances (resistance with a constant value of 1), significant (corrected *P*-value = 0.017) and positive autocorrelation values were only observed in the smaller resistance distance classes (Fig. 2b).

### Univariate and multivariate model optimisation

Landscape resistance as a function of aspect showed the highest correlation coefficient with genetic distance, after removing the effect of the isolation-by-resistance IBR model (Table 1). Land cover and topographic complexity followed aspect respectively (*r* = 0.112, *P* = 0.028; *r* = 0.101, *P* = 0.042; Table 1). Partial Mantel correlations for natural barriers (rivers) and man-made barriers (roads) were not significant, regardless of scale and could therefore be excluded from the remaining analyses (Table 1).

Likewise, when variables were evaluated based on relative support (RS) when compared to IBR and the first step of the causal modelling criteria, the best-supported model was aspect, followed by TC (Table 2). Although roads revealed a positive RS value, this model failed (did not pass) the first step of the causal modelling criteria with IBR. In contrast, land cover and rivers showed negative RS values and did not pass the causal modelling and were therefore not supported (Table 2). Parameter values for aspect were the same as the optimisation based on partial Mantel correlation. The *R*_max_ parameter value for TC was greater than optimisation based on the partial Mantel correlation. Although land cover did not pass the first step of the causal modelling criteria with IBR and RS, this variable was supported based on the partial Mantel correlation (Table 1) and was therefore included in the multivariate analysis. We concluded that the best univariate models were aspect, land cover and TC although only aspect and TC performed significantly better than distance alone.

The best multivariate model based on optimisation with partial Mantel correlation after partialling out the IBR model (see columns A, Table 3) included three variables: aspect, land cover and TC (model 1). Model 2 and 3 were also supported based on the partial Mantel correlation after removing the effect of IBR (Table 3). Model 2 (aspect and land cover), with medium contrast (*x*) and highest resistance (*R*_max_) for aspect and medium contrast (*x*) for land cover, was slightly better supported than model 3 with the same variables.

When model optimisation was based on RS, the best multivariate model comprised aspect, land cover and TC (Table 3, model 1). The RS value improved from 0.018 to 0.053 when TC was included. Model 2 also passed RS but model 3 showed a negative value for RS and was therefore not supported. None of the former models passed the causal modelling, which indicated that none of these models was supported independently of the IBR model (see columns B, Table 3, models 1–3).

In addition, when we applied the causal modelling criteria with the reduced model to model 1, the partial Mantel correlation was significant (*P* = 0.001) when partialling out the effect of the reduced model (GD~(A + L + TC)|(A + L), while the opposite (GD~(A + L)|(A + L + TC) was not significant (*P* = 0.970), indicating that the inclusion of TC improved the model (Table 3). Likewise, aspect passed the causal modelling with the reduced model. However, land cover failed the causal modelling with the reduced model so the only variables that were included in the final multivariate model were aspect and TC (Table 3, model 4). Model 2 also passed the causal modelling with the reduced model. The only supported models in the multivariate framework were A + L and A + TC (Table 3; model 2 and 4). When we compared models 2 and 4 against each other and IBR using RS, model A + TC performed best (see Supplementary Table S3). The RS for model A + TC compared to A + L and IBR was only slightly greater than zero, but it still passed the causal modelling criteria for significance (Table 3). In addition, model A + TC performed significantly better (*r* = 0.113, *P* = 0.026) than distance alone (*r* = 0.003, *P* = 0.485). We concluded that the best multivariate model was A + TC, indicating that aspect influenced gene flow and this pattern is driven by the availability of water and favourable vegetation. Topographic complexity was also likely to influence gene flow.

### Mixed effect models

Statistical models based on resistance distances suggested that environmental variables explained more variation in genetic differentiation than the IBR model that only explained approximately 3% of the variance in genetic distance. The following variables were highly correlated (VIF values > 5) and thus excluded from the linear mixed effect models: TC, IBR, roads and geographic distance. We therefore built a full model (aspect, land cover and rivers) in an attempt to exclude correlated variables. After the inclusion of the remaining landscape variables the amount of variation that has been explained increased to approximately 5% (Table 4). The best-supported model based on the corrected Akaike Information Criterion (AICc; Table 4, model A) included aspect alone (AICc = −12577.60; *w*_*i*_ = 0.88). In general, models within two AIC units of the best-supported model are interchangeable and models with a AICc ≤ ~10 show marginal support. We therefore inferred that the reduced model B (Table 4) that included aspect and land cover (AICc = −12573.60; *w*_*i*_ = 0.11) and model C (aspect and rivers) showed marginal support.

Although model B only showed marginal support based on AICc, this model explained more of the variation (5.2%) in the dependent variable followed by model D that explained 5.1% of the variation (Table 4). Model D comprised aspect, land cover and rivers. In addition, the *R*^2^_*β*_ values of model D (full model) and model B were similar (*R*^2^_*β*_ = 0.051). Likewise, models A (aspect; *R*^2^_*β*_ = 0.046) and C (aspect + rivers; *R*^2^_*β*_ = 0.047) had similar *R*^2^_*β*_ values that explained slightly less of the variation in genetic distance. Based on mixed effect models, we concluded that aspect, land cover and rivers were the variables that explained variation best.

**Figure 3:**
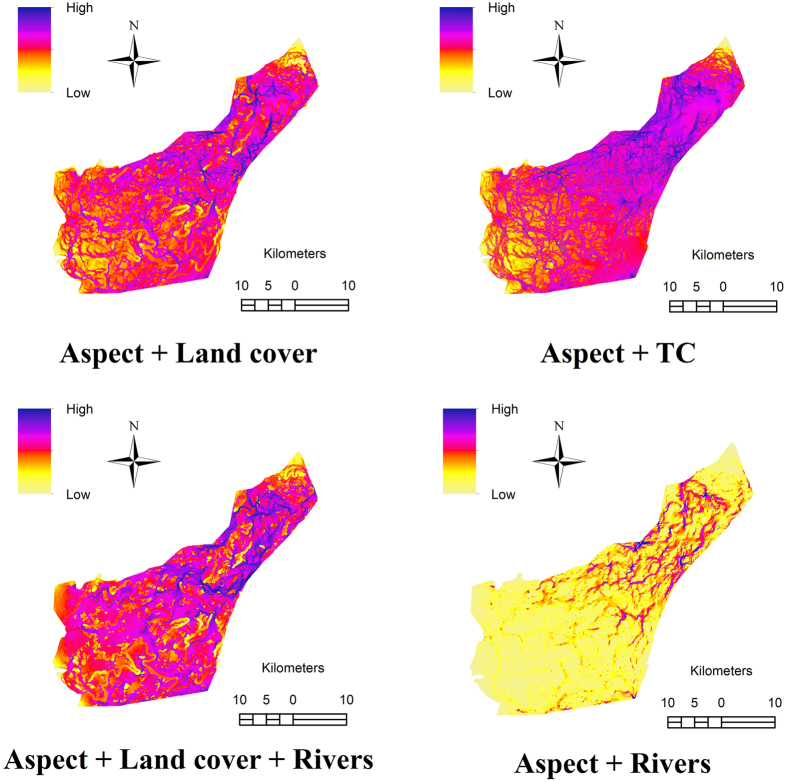
Current maps generated in CIRCUITSCAPE showing connectivity between 101 *Mastomys natalensis* trapping transects from Hluhluwe-iMfolozi Park, South Africa for the following best-supported landscape hypotheses: “Aspect + Land cover”, “Aspect + Topographic complexity”, “Aspect + Land cover + Rivers” and “Aspect + Rivers”. Dark blue represents areas with highest current densities whereas areas with highest resistance (lowest current densities) are represented in the light yellow colour. Areas indicated in dark blue will therefore facilitate gene flow (higher connectivity) whereas areas in light yellow may restrict gene flow. Maps were modified in ArcGIS v 10.1. (http://www.esri.com/software/arcgis/arcgis-for-desktop).

Based on our best-supported multivariate hypotheses, the most likely regions of genetic connectivity for *M. natalensis* are represented in Fig. 3. Areas with highest resistance to gene flow are indicated in light yellow. All maps showed high spatial heterogeneity in connectivity with a north to south gradient of high to low connectivity except for map “Aspect + Rivers”. Eastern aspect and land cover presented the least resistance to dispersal/movement through the landscape for this small mammal. Map “Aspect + Rivers” showed that the rivers in the park exhibit low current density (restriction to gene flow). This map also showed no clear north to south gradient of high to low connectivity but rather displayed high landscape connectivity in the northern part of the park.

## Discussion

We present the first landscape genetics study for an African small mammal, identifying features that may potentially explain contemporary genetic structure in a relatively unmodified landscape. Our results showed a higher and significant genetic correlation among individuals than expected at smaller geographic scales (up to about 11 km) confirming the results of van Hooft *et al*.^3^ suggesting that *M. natalensis* exhibit a pattern of kin clustering at smaller geographic scales while, long distance dispersal events result in no pattern of IBD as suggested by spatial autocorrelation at larger geographic scales. It has also been documented that if animals breed at a young age that they will show a greater tendency to breed close to their natal site^14^. This further supports the pattern of IBD as indicated by spatial autocorrelation at smaller geographic scales. Long distance dispersal will erode IBD patterns and therefore weak patterns of IBD could be due to rare long distance dispersal as we report in this study for *M. natalensis* and as has been found for other small mammals^26^.

Low to moderate differentiation indicates a pattern of sub-clustering of up to four potentially relevant clusters that differ in their geographic coherence/genomic integrity that does not coincide with those expected if divergence was driven solely by isolation-by-distance (all *F*_ST_ values were significant and ranged from 0.025–0.124 between clusters). The most closely related individuals are not necessarily those that come from the same geographical area. This indicates that forces imposing population genetic structuring are not based purely on IBD as suggested by spatial autocorrelation. Rather, movement seems to be restricted providing support for the hypothesis that restricted dispersal is one of the forces driving patterns of genetic structure in *M. natalensis*. This has also been evident in other landscape genetic studies of rodent species^27^. Complex and inconsistent patterns of genetic heterogeneity have been described as “chaotic genetic patchiness” consistent with the patterns observed in this study^28^ and the landscape features investigated in this study may underlie this pattern.

In the broader sense, a landscape genetics approach should be incorporated into spatial models of disease processes to increase the ability to predict patterns of disease occurrence, to prevent the spread of disease and to develop management policies^29^. Likewise, the identification of barriers to movement in mammal disease reservoirs is important as this can assist in disease containment strategies^30^, pest and crop management^31^.

The results of the univariate/multivariate parameter optimisation suggest that slope aspect is the landscape variable most strongly correlated with gene flow in *M. natalensis*, followed by land cover, topographic complexity (TC) and rivers. Slope aspect promotes gene flow in *M. natalensis*, with western facing slopes posing greater resistance than eastern facing slopes. Similarly, Castillo *et al*.^32^ provided evidence that eastern facing slopes are positively correlated with genetic connectivity in the American pika. Land cover is also strongly correlated with gene flow in *M. natalensis* with thicket vegetation as the most favourable. TC and rivers (movement across rivers) are the two landscape features that most strongly restrict gene flow. This suggests that *M. natalensis* dispersal is mainly restricted by physical limitations. Complex topographies such as ridges and steep slopes pose more resistance to movement. Resistance to gene flow increases as a landscape becomes more complex because of greater energetic cost to movement due to the small body size of *M. natalensis*. Rivers/streams may act as barriers to gene flow as is evident from the Bayesian clustering analysis (Cluster I). Although not explicitly tested in this study, the size/width of the watercourse/river is also likely to determine the extent to which a barrier is permeable. Having stated this, there may or may not be facilitation of gene flow along rivers. In general, rivers appear to be barriers to gene flow but it is evident from this study that *M. natalensis* are able to traverse these barriers confirming their swimming behaviour as described by Hickman and Machiné^11^. Watercourses have shown to have a small effect on gene flow and may impede movement but it does not act as a complete barrier to gene flow. Likewise, it has been shown that migration between small mammal sub-populations occurs across rivers^27^.

*Mastomys natalensis* is commonly found across a large range of different habitats except deserts and very high altitudes^24^. Although classified as a generalist, some landscape features may still have an influence on gene flow in this small mammal. A number of studies have suggested that landscape features can be prominent in shaping genetic structure in generalist species. For example, it has been shown that a river and a motorway represented a barrier to dispersal in the Eurasian badger (*Meles meles*)^33^. Genetic differentiation for habitat specialists is expected to be larger than for generalist species^34^. Here, we identify aspect and the associated vegetation cover as important landscape features in shaping the genetic structure in this generalist species.

Due to the fact that this park is in the southern hemisphere, western facing slopes represent a heterogeneous dry tropical savanna habitat. In contrast, the eastern facing slopes are characterised by a mesic and cooler habitat (lower solar radiation) with dominant vegetation featuring wooded trees^35^. Eastern facing slopes are in general cooler (lower temperatures because of morning sun) whereas western facing slopes experience higher afternoon temperatures. In addition, southern/eastern facing slopes in the region tend to desiccate more slowly than the northern/western facing slopes (G Clinning, personal communication, Ezemvelo KZN Wildlife).

South Africa is in general characterised by a decrease in rainfall from east to west and the eastern regions of the coastal plateau are more humid^36^. Southern/eastern facing slopes are therefore wetter in general than northern/western facing slopes because moisture and air masses originate from the Indian Ocean (see Supplementary Fig. S5)^36^. These air masses cause the trapping of rain that moves inland^37^. This area of South Africa is also known as the “mist belt”; the mist is usually associated with southerly winds providing moisture to eastern facing slopes^36^. This higher rainfall provides suitable conditions to sustain thicket vegetation however, there may be dry periods that prevent the development of forest^38^. Thicket is renowned for its high biodiversity and includes evergreen, sclerophyllous or succulent trees, shrubs and vines^38^. Thicket often comprises dense impenetrable vegetation (shrubs) with no distinct layer of trees and shrubs^38^, providing small mammals with cover and protection against predators while foraging and dispersing. In addition, thicket may provide a greater variety of food sources such as fruits and vegetative plant material, i.e. leaves, stems and seeds^10^. The apparent preference of *M. natalensis* for eastern facing slopes (areas of higher rainfall) and thicket vegetation (areas of seasonally available water) demonstrate the species’ dependency on seasonally available water^10^.

Dispersal, resource availability and population densities affect genetic structure in natural populations^39^ but landscape heterogeneity may also promote/facilitate gene flow between populations. Patterns of genetic sub-structuring observed in this study may be explained by a combination of shorter-distance movements (when population densities are low) with increasing distances when population densities increase^40^.

We used a range of analytical and modelling approaches, all with some limitations. Mantel and partial Mantel tests have been widely used in landscape genetic studies^41^,^42^ although some authors have debated the use of these tests due to the potential increase in Type I error^43^. Error can be avoided by evaluating the correlation coefficient itself as opposed to the test for significance, an approach that may explain why landscape variables such as rivers and land cover are not supported in the present study using the partial Mantel framework. In addition, the effect of rivers on gene flow was not strong enough to be detected in the partial Mantel tests. We therefore implemented mixed effect models on distance matrices since analysing data using only one method could result in false, method-dependent outcomes^43^.

In light of global warming, the dry regions of the subtropics are likely to get drier which will result in less cover and an absence of thicket vegetation. As such, population connectivity of *M. natalensis* may be affected by climate change. By using *M. natalensis* in a landscape genetics context, one can expand the results to the ecosystem management of other small mammal species with similar ecosystem functions. In addition, apart from contributing to landscape genetic studies in Africa^21^ the present study may have implications in epidemiological and agricultural research associated with problem rodents.

## Methods

### Study site, sampling and genotyping

**Figure 4:**
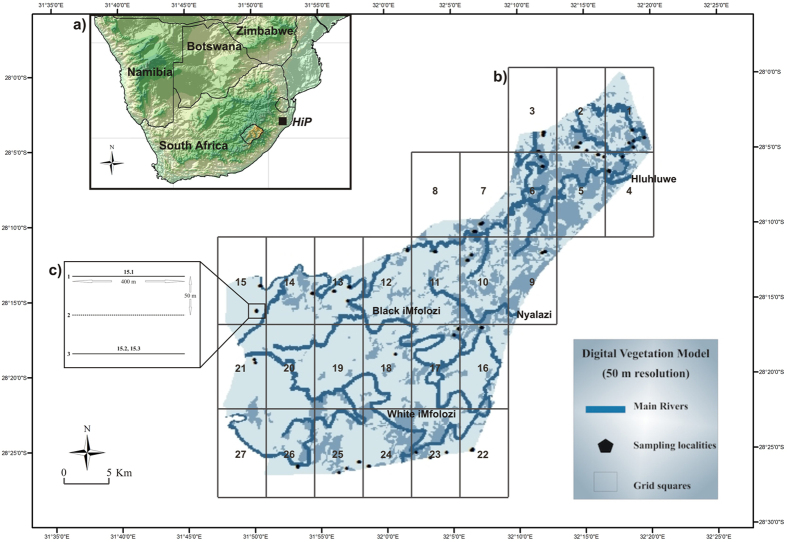
Map of the Hluhluwe-iMfolozi Park (HiP) situated in the KwaZulu-Natal province (see map (**b**)) of South Africa (see map (**a**) indicated by black square). The park has been divided into 27 grids and samples were collected to represent all habitat types. Sampling coordinates along each transect within a grid were combined into a midpoint coordinate for that transect. Main rivers (in blue) and thicket vegetation (in light blue-grey) are also indicated on the map. Inset (**c**) shows a schematic diagram of transect layout for grid 15. This map was modified in CorelDraw Graphics Suite X3 (2014; http://www.coreldraw.com).

Hluhluwe-iMfolozi Park (HiP) is situated in the northern escarpment of the KwaZulu-Natal province of South Africa, covering an area of approximately 960 km^2^ (28°17’49”S; 31°44’32”E) and contains four large rivers: the Hluhluwe, Nyalazi, Black and White iMfolozi Rivers (Fig. 4)^44^. The climate is coastally modified and varies according to topography with a mean annual rainfall of 985 mm at higher altitude^44^. Annual temperatures range from 13 °C to 35 °C and the park is situated in the Savanna biome of South Africa, supporting a variety of habitat types from scrap forest to thicket^38^.

Rodent samples were collected using a landscape grid (see Supplementary Table S1). All permit information was reported (see Supplementary Table S2). The sampling method was carried out in accordance with Ezemvelo KZN Wildlife protocols and animals were handled under the guidelines of the American Society of Mammalogists (ASM; Animal Care and Use Committee, 2011)^45^. Ear biopsy sampling received ethical approval from Cardiff University in September 2010.

The park was divided into 27 sections, each being approximately 10 km North-South × 5 km East-West (Fig. 4). One of the sections (8) was not sampled since it only included a small part of the park and was inaccessible (Fig. 4). No *M. natalensis* could be captured from three (section 19, 20 and 27) of the remaining 26 sections. We therefore sampled 23 sections across the park. A total of 150 Sherman traps (H.B. Sherman Traps Inc. Florida, USA) were placed in each section under logs, rocks and tussocks in an attempt to stratify sampling across the landscape. Sampling was carried out up to a maximum of four nights per section, by using three transects per night approximately 50 m apart (~400 (l) × 100 (w) m), with a 10–15 m inter-trap interval (Fig. 4, inset c). Up to 21 individuals were collected per section over a total of 4,516 trap nights, resulting in a total of 272 samples, 260 of which were analysed; twelve being excluded because of poor amplification success rate. Tissue samples for DNA analysis were collected by ear biopsy and stored in 99% ethanol at −20 °C.

Total genomic DNA was extracted from tissue using a DNeasy^®^ Tissue Kit (QIAGEN^®^ Hilden, Germany) following the manufacturer’s instructions. Genotyping was carried out using 10 microsatellite markers originally isolated from *Mastomys huberti*^46^,^47^. We followed PCR conditions as described in Galen *et al*.^46^ and Loiseau *et al*.^47^. Amplifications were carried out in a total volume of 10 μl containing 5 μl of QIAGEN Multiplex PCR Master Mix, 0.2 μM of each of the forward and reverse primers and 1 μl of DNA (~50 to 100 ng/μl). The ten primer pairs were divided into three multiplexes and one singleplex using three dyes (FAM, HEX and TAM): MH28, MH30, MH60, MH133, MH141 (Multiplex 1); MH05, MH74 (Multiplex 2); MH105, MH188 (Multiplex 3) and MH80 (Singleplex 1). PCR products were processed commercially by Macrogen Inc, Korea (www.macrogen.com/eng/). Electropherograms were scored using GeneMarker v 1.91 (SoftGenetics LLC). Observed allele lengths were binned where necessary and rounded to integers using the software TANDEM v 1.8^48^.

### Statistical analyses

We checked for the presence of null alleles using MICROCHECKER^49^. The rarefaction procedure implemented in FSTAT v 2.9.3.2^50^ was used to estimate the expected number of alleles and to compare allelic richness (*r*). Hardy-Weinberg equilibrium (HWE) was analysed for each locus using FSTAT v 2.9.3.2^50^. Linkage disequilibrium (LD) between all pairs of loci and *F*-statistics between clusters were estimated using GENEPOP v 4.1^51^. Statistical significance for HWE and LD was assessed by *P*-values and the implementation of the modified false discovery rate (FDR) method^52^ for multiple tests. Observed (*H*_O_) and unbiased expected (_*U*_*H*_E_) heterozygosities were estimated for all loci using GENETIX v 4.5.0.2^53^.

### Population structure

Bayesian clustering in STRUCTURE v 2.2.3^54^ was used to infer the most likely number of population clusters (*K*). The analysis was implemented for 450 000 iterations following a burn-in of 50 000 iterations with no *a priori* locality data. Both the posterior probability of the data for the given value of *K* (LnPr(*X*|*K*)) and its rate of change (∆*K*)^25^ were used to evaluate population structure. Twenty independent runs were carried out for *K* values from one to ten and an admixture model with correlated allele frequencies^54^ was assumed. Progressive partitioning at various hierarchical levels using *K* = 2^55^ was used to further examine structure within resolved clusters.

### Genetic distance and spatial autocorrelation

We analysed the genetic data (a reduced dataset of 216 individuals) using transect lines as the unit of spatial analysis. The geographical midpoints of each transect were used as location coordinates (see Supplementary Table S1). We estimated pairwise genetic distance for each transect by using the proportion of shared alleles in MSA v 4.05^56^ (Table S4). Euclidean geographic distances in kilometres were calculated between all transects and pairwise resistance distances were estimated using CIRCUITSCAPE v 3.5^57^ by creating a raster file with a constant value of 1 for all pixels (isolation-by-resistance (IBR) model).

A Mantel correlogram using the per transect association between the proportion of shared alleles and geographic or resistance distances (*r*_M_) was calculated to test for spatial autocorrelation using a permutation test of significance (10 000 iterations). The modified false discovery rate method^52^ was employed to correct for global significance.

### Landscape resistance optimisation (univariate and multivariate models)

Landscape resistance was modelled as a function of aspect, river, roads, topographic complexity (TC) and land cover according to a landscape resistance hypothesis (see Table 5 for summary). Optimised values included equation parameters for *x* (contrast) and *R*_max_ (magnitude of the relationship). For a full description see the Supplementary information. Pairwise landscape resistance matrices for each resistance surface were estimated using CIRCUITSCAPE v 3.5^57^ by implementing the pairwise mode option with focal points, connecting eight neighbours based on the average resistance.

Partial Mantel tests have been widely used in landscape genetic studies although some authors have debated the use of these tests^58^. Partial Mantel tests can be successful when used in the causal modelling framework as was done in this study^41^.

Genetic distance was compared with landscape resistances to identify the functional scale for which each variable best explained the observed genetic structure. Scaled transformations for each landscape variable based on a unimodal peak of support^59^ in the partial Mantel correlation coefficient were used to rank variables/models. All statistical analyses were performed in R^60^.

### Univariate and multivariate model selection

We used a combination of causal modelling approaches proposed by Cushman *et al*.^58^ and Wasserman *et al*.^61^. The first step^58^,^61^ suggests that if a resistance hypothesis is supported independently of the null model then: (A) partial Mantel tests between the genetic distance and landscape variable would be significant after removing the effect of IBR (GD~LV|IBR); (B) partial Mantel tests between genetic distance and IBR distance would not be significant, partialling out the landscape variable (GD~IBR|LV). The second step^58^ allows for the comparison of causal modelling with a reduced model. If a landscape model (true model) is supported independently of the other candidate models then: (C) partial Mantel tests between genetic distance and the landscape model would be significant, removing the effect of the reduced model (GD~LM|); (D) partial Mantel tests between genetic distance and the reduced model would not be significant, partialling out the effect of the landscape model (GD~|LM).

Candidate models with similar parameters to the top model were assessed against each other rather than simply against IBR to evaluate models based on relative support (RS). Relative support can be defined as: RS_A|B_ = (GD~A|B)–(GD~B|A) where GD is the genetic distance, A is the resistance distance matrix for variable A, B is the resistance matrix obtained for variable B and (GD~A|B) is the partial Mantel *r* between GD and variable A after removing the effect of variable B. The landscape model with a positive RS in all comparisons represents the best model^58^. In order for landscape models to be accepted, models also had to pass the causal modelling criteria with IBR.

Landscapes are complex structures and therefore best described using multivariate approaches that incorporate multiple landscape features. Here, we built rasters equal to the sum of the univariate rasters for each landscape variable. The two landscape variables with the highest partial Mantel *r* when removing the effect of IBR were used to create a series of bivariate models by keeping the parameters constant for the first variable, while varying the parameters for the second variable. The best-supported model for the second variable was identified based on the partial Mantel correlation removing the effect of IBR. Subsequently, we identified the optimum parameters for the first variable by holding the second constant. Once we identified the optimum parameters for the first two models, additional landscape variables were added while keeping the first two model parameters constant. We re-optimised the remaining variables until the best-supported model did not change. All models were also evaluated using their RS by varying the model parameters for one variable while holding the others constant until variable parameters stabilised. Models were required to pass the two causal modelling steps and variables were also required to pass the causal modelling criteria with the reduced model. For example, in order for variable C to be accepted the following needs to be true for the different multivariate rasters: GD~(A + B + C)|(A + B) must be significant and GD~(A + B)|(A + B + C) must be non-significant. Multivariate rasters (A + B + C; A + B) contained the combined information of variables A, B and C. Partial Mantel tests were performed in R using the “vegan” package^62^.

### Mixed effect models

In addition to causal modelling, we also implemented linear mixed effect models in R using the “lme4” package^63^ to account for dependency between pairwise observations in a distance matrix^64^. To correct for the dependency among pairwise data points in our data, we followed the maximum likelihood populations-effects (MLPE) method as described in Clarke *et al*.^65^ and more recently in Van Strien *et al*.^66^. Sampling unit was introduced as a random effect term that accounted for the non-independency of pairwise sampling unit distances by assuming an intercept that was different for each unit. The random effect term in the model specified the pairwise sampling unit structure of the dataset. In addition, all explanatory variables were introduced as fixed effect terms. Restricted maximum likelihood (REML) estimates of the intercept were the same as for those obtained from linear regression^65^ because all explanatory variables were standardised. In each model, the random effect was standard with different explanatory variables. First we calculated the Variance Inflation Factors (VIF) for each variable in the model^67^. Variance Inflation Factors values above 5 show evidence for colinearity and explanatory variables exhibiting significant levels of multi-colinearity were removed from the models to minimise potential error^68^.

We built a full model with all variables to identify the combined effects of multiple variables on gene flow. This model only included the variables that did not show any evidence of colinearity. The full model was refined by using multi-model inference in the “MuMIN” package^69^ of R. Maximum-likelihood populations-effects (MLPE) models were fitted with REML estimation to account for the association of each pairwise distance between sampling units. This method is desirable for unbiased estimates of the variance components of mixed models^65^. We used model averaging from a global model to select the best models by calculating the corrected Akaike Information Criterion values (AICc)^70^. Akaike Information Criterion scores are often represented as ΔAICc scores, which indicates the difference between the best model (ΔAICc = 0) and all the other candidate models. We also calculated AICc weights (*w*_*i*_). Models with the lowest change in the AICc score (ΔAICc = 0) and the highest Akaike weight were considered the best^71^. Models within two AIC units of the best-supported model were interchangeable and models with a ΔAICc ≤ 10 showed marginal support^70^.

In addition, we calculated the *R*^2^_*β*_ statistic^72^, which compared a model with fixed effects to a null model (we used the IBR model as the null model). The null model comprised of the random effect and an intercept. The *R*^2^_*β*_ was calculated from the Kenward-Rodger *F* and degrees of freedom^73^. We used the “KRmodcomp” function from the R package “pbkrtest”^74^. We used the *R*^2^_*β*_ statistic to describe the proportion of variation being explained by the models.

### Current maps

Current maps based on our best-supported multivariate hypotheses were generated in CIRCUITSCAPE v 3.5^57^ in order to indicate the most likely regions of genetic connectivity/movement for *M. natalensis* within the Hluhluwe-iMfolozi Park.

### Data accessibility.

Microsatellite data and GIS raster maps available from the Dryad Digital Repository: http://dx.doi.org/10.5061/dryad.492b8.

## Additional Information

**How to cite this article**: Russo, I. M. *et al*. Landscape determinants of fine-scale genetic structure of a small rodent in a heterogeneous landscape (Hluhluwe-iMfolozi Park, South Africa). *Sci. Rep.* **6**, 29168; doi: 10.1038/srep29168 (2016).

## Supplementary Material

Supplementary Information

Supplementary Table

## Figures and Tables

**Table 1:** The best univariate models of effective landscape resistances based on partial Mantel correlation after removing the effect of the isolation-by-resistance (IBR) model.

Landscape variable	Parameter values	Partial Mantel *r*	*P*-value
Aspect	90°; *x* = 10; *R*_max_ = 2	***0.116***	***0.024***
Land cover	*x* = 1; *R*_max_ = 1 000	***0.112***	***0.028***
Topographic complexity (TC)	*x* = 10; *R*_max_ = 2	***0.101***	***0.042***
Rivers	Classified; *R*_max_ = 5 000	*0.036*	*0.254*
Roads	Classified; *R*_max_ = 2	*0.030*	*0.289*

Models are ranked according to the Partial Mantel *r*-value. Optimised parameter values, partial Mantel *r* and significance of support are shown. Supported models are indicated in bold.

**Table 2:** The best univariate models based on relative support (RS) and causal modelling after removing the effect of the isolation-by-resistance (IBR) model.

Landscape variable	Parameter values	RS_IBR_	(A) *r*	(A) *P*	(B) *r*	(B) *P*	Supported
Aspect	90°; *x* = 10;*R*_max_ = 2	***0.168***	***0.116***	***0.023***	***−0.052***	***0.832***	**Yes**
Topographic complexity	*x* = 10;*R*_max_ = 10	***0.094***	***0.097***	***0.050***	***0.003***	***0.500***	**Yes**
Land cover	*x* = 0.5;*R*_max_ = 1 000	*−0.012*	*0.112*	*0.029*	*0.124*	*0.0001*	No
Rivers	Classified;*R*_max_ = 5	*−0.024*	*0.032*	*0.278*	*0.056*	*0.139*	No
Roads	Classified;*R*_max_ = 2	*0.021*	*0.030*	*0.291*	*0.009*	*0.440*	No

Models are ranked with the best-supported model at the top. Optimised parameter values, RS as compared to IBR, partial Mantel *r* and significance of support are shown. Optimised values include equation parameters for *x* (contrast) and *R*_max_ (magnitude of the relationship). (A) GD~LV|IBR - partial Mantel test between genetic distance and the landscape variable, partialling out the effect of IBR; (B) GD~IBR|LV - partial Mantel test between genetic distance and IBR distance, removing the effect of the landscape variable. The first column of each test indicates the Mantel *r*-value and the second column the related *P*-value. Supported models are indicated in bold.

**Table 3:** The best multivariate models based on relative support (RS), causal modelling after removing the effect of the isolation-by-resistance (IBR) model (A,B) and causal modelling criteria with the reduced model (C,D).

	Model	Parameters	RS_IBR_	(A) *r*	(A) *P*	(B) *r*	(B) *P*	(C) *r*	(C) *P*	(D) *r*	(D) *P*
1)	A + L + TC	*A*: 225°; *x* = 4;*R*_max_ = 1 000	*0.053*	*0.127*	*0.016*	*0.074*	*0.023*	*A: 0.124*	*0.004*	*A: −0.059*	*0.891*
		*L: x* = 2;*R*_max_ = 1 000						*L: 0.086*	*0.071*	*L: 0.096*	*0.014*
		*TC: x* = 4;*R*_max_ = 1 000						*TC: 0.110*	*0.001*	*TC: −0.073*	*0.970*
2)	A + L	*A*: 225°; *x* = 4;*R*_max_ = 1 000	*0.018*	*0.124*	*0.022*	*0.106*	*0.0008*	*A: 0.144*	*0.002*	*A: −0.087*	*0.966*
		*L: x* = 2;*R*_max_ = 1 000						*L: 0.102*	*0.042*	*L: 0.081*	*0.064*
3)	A + L	*A*: 225°; *x* = 10;*R*_max_ = 2	*−0.033*	*0.115*	*0.029*	*0.148*	*0.0001*	*–*	*–*	*–*	*–*
		*L: x* = 1;*R*_max_ = 1 000						*–*	*–*	*–*	*–*
4)	A + TC	*A*: 225°; *x* = 4;*R*_max_ = 1 000	*0.110*	*0.113*	*0.026*	*0.003*	*0.485*	*A: 0.113*	*0.0001*	*A: −0.018*	*0.630*
		*TC: x* = 4;*R*_max_ = 1 000						*TC: 0.176*	*0.0001*	*TC: 0.050*	*0.187*

Optimised parameter values, RS as compared to IBR, partial Mantel *r* and significance of support are shown. Optimised values include equation parameters for *x* (contrast) and *R*_max_ (magnitude of the relationship). (A) GD~LV|IBR - partial Mantel test between genetic distance and the landscape variable, partialling out the effect of IBR; (B) GD~IBR|LV - partial Mantel test between genetic distance and IBR distance, removing the effect of the landscape variable, (C) GD~LM| - partial Mantel test between genetic distance and the landscape model after removing the effect of the reduced model; (D) G~|LM - partial Mantel test between genetic distance and the reduced model, partialling out the effect of the landscape model. The first column of each test indicates the Mantel *r*-value and the second column the related *P*-value. Model abbreviations: A = Aspect; L = Land cover and TC = Topographic complexity.

**Table 4:** Mixed effect models showing the relationship between pairwise genetic distances and resistance distances for different environmental variables.

Model	Type of model	Variables	*R*^2^_*β*_	VIF	AICc	∆AICc	Weight (*w*_*i*_)
A	Reduced	Aspect	0.046	3.12	−12577.60	**0.00**	0.88
B		Aspect	0.052	3.12	−12573.60	**4.03**	0.11
		Land cover		1.69			
C		Aspect	0.047	3.12	−12565.70	11.86	0.01
		Rivers		1.54			
D	Full	Aspect	0.051	3.12	−12563.00	14.59	0.00
		Land cover		1.69			
		Rivers		1.54			

In order to minimise colinearity among predictors, all variables with VIF values > 5 were removed. VIF = Variance Inflation Factor. The best fitting model was selected using the corrected Akaike Information Criterion (AICc, ∆AICc, *w*_*i*_). We used *R*^*2*^ statistics (*R*^*2*^_*β*_) to describe the amount of variation explained by the model. Models with the highest AICc support are in bold (∆AICc ≤ 2). Marginally supported models are also indicated (∆AICc ≤ 10).

**Table 5:** A summary of the landscape variables and the corresponding resistance hypotheses.

Variable	Hypothesis	Parameters	Maps
Aspect	The optimal aspect (less resistance) is associated with the availability of water and favorable vegetation	*x* = 0.5, 1, 2, 4, 10	200
		*R*_max_ = 2, 10, 100, 500, 1 000	
		*θ*_*opt*_ = 0, 45, 90, 135, 180, 225, 275, 315, 360	
		*θ* = 0, 45, 90, 135, 180, 225, 275, 315, 360	
		Flat areas = *R*_max_/2	
Rivers	Physical barrier to small mammal movement	*R*_max_ = 2, 5 10, 50, 100, 250, 500, 750, 1 000, 5 000, 10 000	22
		Land = 1	
Roads	Physical barrier to small mammal movement	*R*_max_ = 2, 5 10, 50, 100, 250, 500, 750, 1 000, 5 000, 10 000	22
		Land = 1	
TC	Resistance to gene flow increases as landscape becomes more complex	*x* = 0.5, 1, 2, 4, 10	150
		*R*_max_ = 2, 10, 100, 500, 1 000	
		Radii = 1, 2, 5, 10, 25, 50	
Land cover	Land cover as a source of food and cover against predators promotes gene flow	Array of resistance values (permutations) =1, 168, 334, 501, 667, 1 000	120
		*R*_FH_ = 1; *R*_max_ = 2, 5, 10, 50, 100, 250, 500, 750, 1 000, 5 000, 10 000, 100 000	36
		*x* = 0.5, 1, 2, 4, 10; *R*_min_ = 1; *R*_max_ = 2, 5, 10, 50, 100, 250, 500, 750, 1 000	180

Parameter values for each variable are indicated. These include *x* (power function), *R*min/*R*max(minimum or maximum resistance), *θ*_*opt*_. (hypothesised optimal aspect in increments of 45° from 0° to 315°), *θ* (aspect value in increments of 45° from 0° to 315°), radii (buffer area in number of cells) and *R*_FH_ (favourable habitat). Abbreviations: TC = Topographic complexity. The total number of maps generated for each variable are indicated in the last column. For a full description see the Supplementary information.
