# Supplementary material for: Landscape determinants of fine-scale genetic structure of a small rodent in a heterogeneous landscape (Hluhluwe-iMfolozi Park, South Africa)
Source: Sci Rep. 2016 Jul 13;6:29168. doi: 10.1038/srep29168 (PMC4942783; doi:10.1038/srep29168)
Supplement: Supplementary Information [file srep29168-s1.pdf]

SUPPLEMENTARY INFORMATION

Landscape determinants of fine-scale genetic structure of a small rodent in a heterogeneous landscape (Hluhluwe-iMfolozi Park, South Africa)

Isa-Rita M. Russo, Catherine L. Sole, Mario Barbato, Ullrich von Bramann, Michael W. Bruford

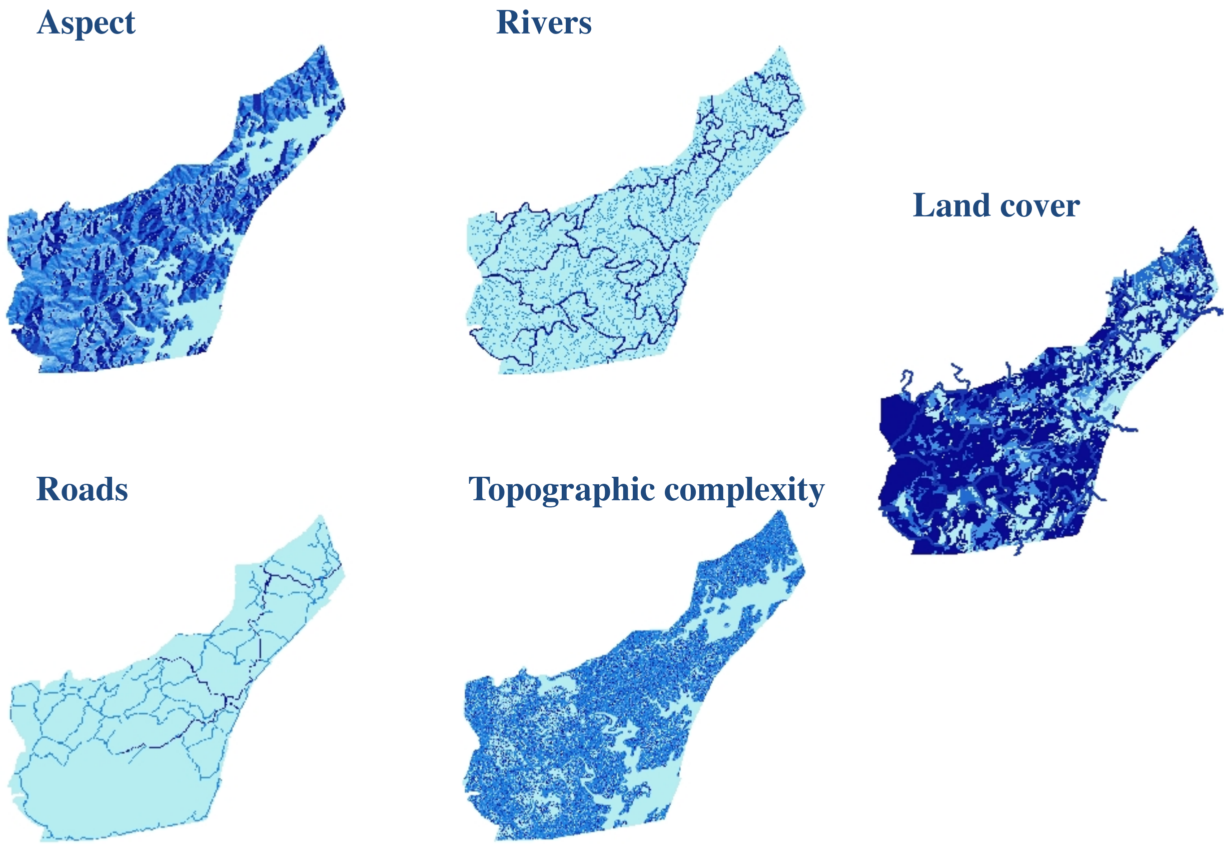

Figure S1 Raster maps provided by HiP (Hluhluwe-iMfolozi Park) for the different landscape variables: aspect, rivers, roads, topographic complexity (TC) and land cover. All baseline maps were provided by Geoff Clinning (Ezemvelo KZN Wildlife, Hluhluwe Research Centre, Geographic Information Systems Unit) and maps were modified in ArcGIS v 10.1 (http://www.esri.com/software/arcgis/arcgis-for-desktop).

Equations

Equation S1: Resistance as a function of aspect modified for the southern hemisphere

$$R = \left[\frac{1 - \cos\left(\left|180 - \left|\theta - \theta_{opt} - 315\right|\right|\right)}{2}\right]^{x} R_{max} + 1$$

where θ is the aspect value in degrees of interest, θ_{opt} is the hypothesised optimal aspect such that resistance increases towards R_{max} (θ = 0° or 360°) according to a curve governed by a power function (x). Parameter values tested are as follows: x = 0.5, 1, 2, 4, and 10; R_{max} = 2, 10, 100, 500, and 1 000. Pixels with a value of -1 (flat areas) were reclassified as $Rmax/2$.

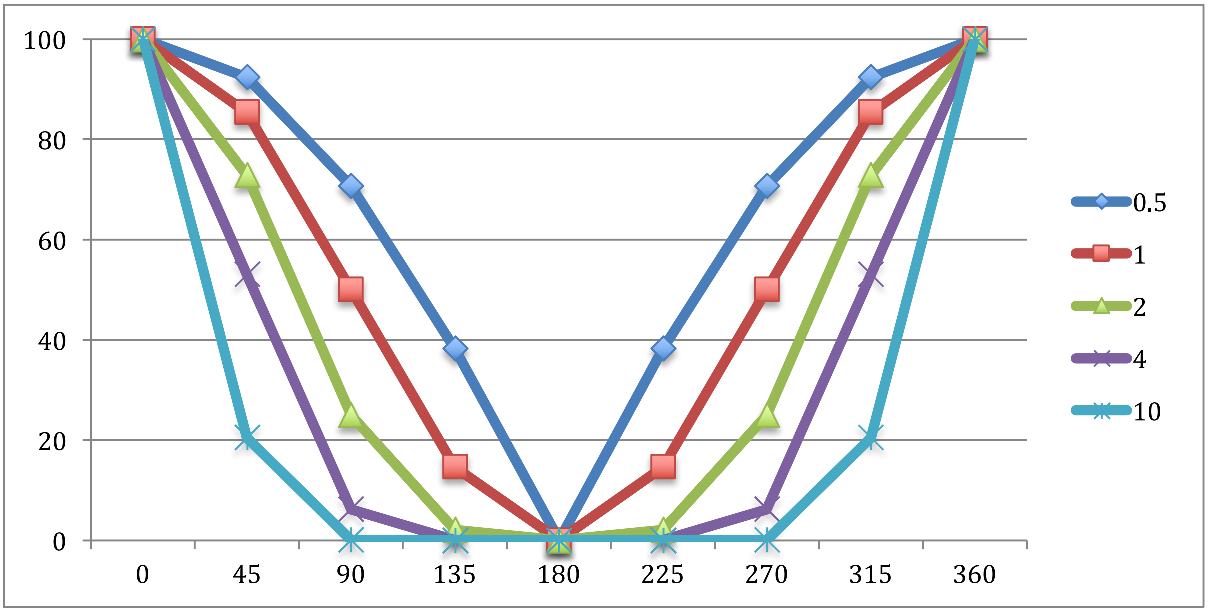

Figure S2 Resistance hypothesis for aspect with an optimal aspect of 45° and an R_{max} of 1 000 for the following power functions (x): 0.5, 1, 2, 4, and 10. The x-axis indicates different aspect values in degrees (θ = 0° - 360°) and the y-axis indicates resistance values (0 - 100) as a percentage.

Equation S2: Resistance as a function of topographic complexity (TC)

$$R = (SRR)^x R_{max} + 1$$

Six different radii were used (1, 2, 5, 10, 25, and 50) to calculate surface relief ratio (SRR). Values of 0 and 1 indicated low and high topographic complexity, respectively. Parameter values tested are as follows: $x = 0.5, 1, 2, 4,$ and 10; $R_{\text{max}} = 2, 10, 100, 500,$ and 1 000.

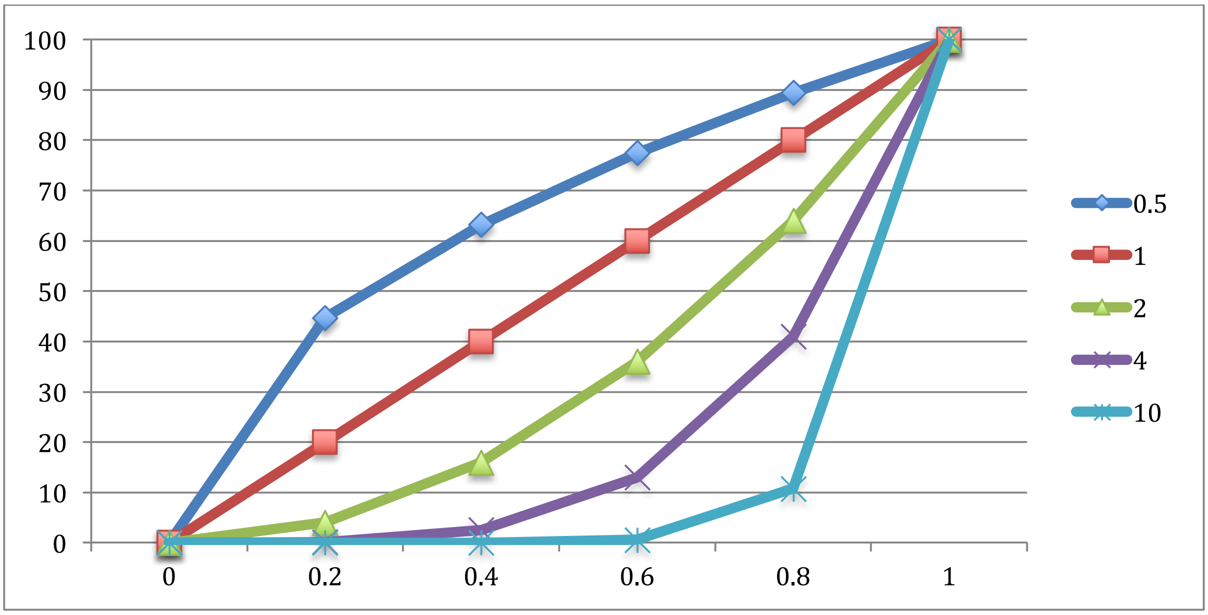

Figure S3 Resistance hypothesis for topographic complexity (TC) with an R_{max} of 1 000 for the following power functions (x): 0.5, 1, 2, 4, and 10. The x-axis indicates different topographic complexity values (0 - 1) and the y-axis indicates resistance values (0 - 100) as a percentage.

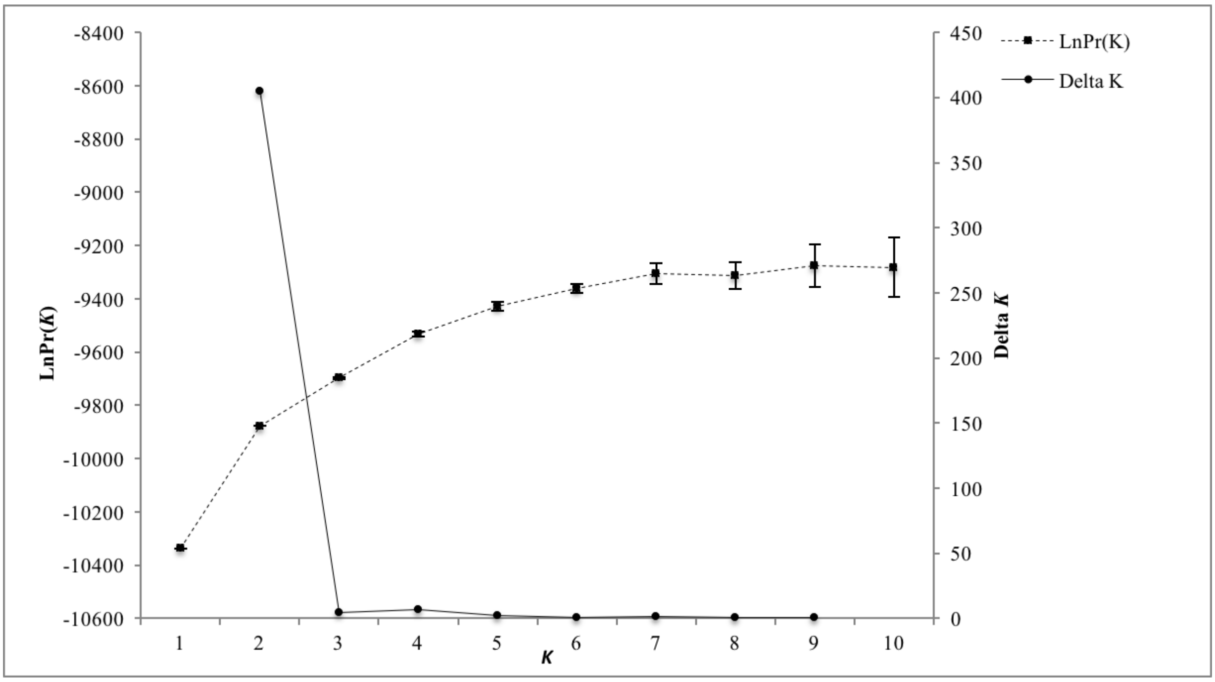

Figure S4 Estimated number of clusters inferred using Bayesian clustering analysis performed with STRUCTURE for the whole dataset. Black squares indicate the probability log-likelihood from 20 repeats for each assumed value of *K*. Black circles indicates values of the statistics ΔK. ΔK tends to peak at the K = 2 followed by K = 4. Bars represent standard deviation.

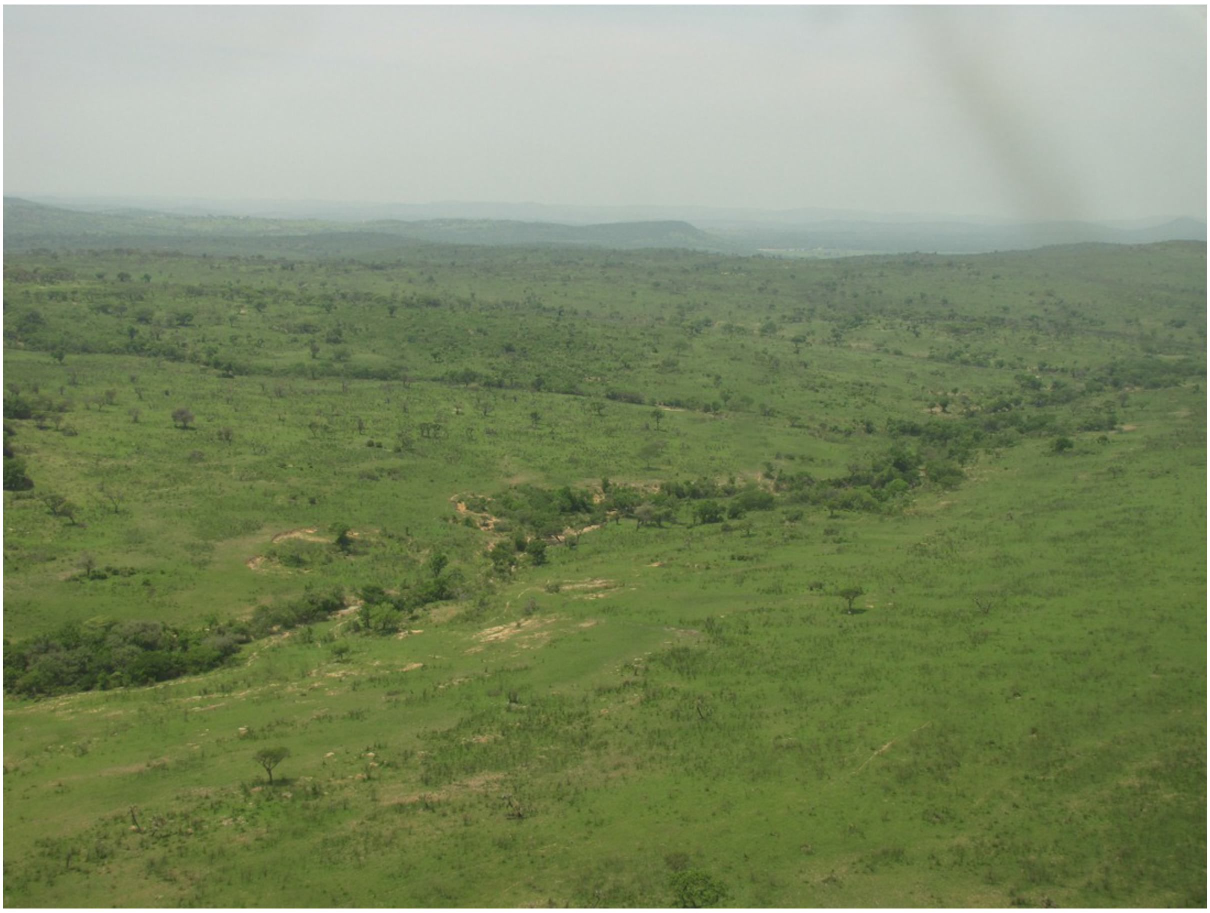

Figure S5 A photo showing the differences between southern and northern facing slopes in the Hluhluwe-iMfolozi Park, South Africa. The top left above the drainage line indicates the southern facing slopes, typically characterised by wooded trees providing more cover to small mammals. In contrast, the northern facing slopes (bottom right below the drainage line) are characterised by fewer trees. Credit: Geoff Clinning.

Table S1 Summary of collection sites indicating the sampling grid number (corresponding to numbers in Fig. 4), transect number, number of samples (*N*) and the midpoint geographic coordinates for each transect for a total of 216 *Mastomys natalensis* individuals from the Hluhluwe-iMfolozi Park that were used in the landscape analyses. This table refers to the samples used in the landscape and spatial autocorrelation analyses. See Dryad Digital Repository for this reduced and the full dataset.

Sample number	Grid number	Transect number	*N*	Latitude	Longitude
1	**1**	1	1	32.12222	28.07889
2		2	2	32.12611	28.06556
3		3	3	32.12556	28.06556
4		4	1	32.12722	28.07694
5		5	1	32.12722	28.08333
6		6	1	32.12722	28.08389
7		7	2	32.13889	28.07278
8		8	3	32.13889	28.07306
9		9	3	32.13889	28.07361
10	**2**	10	1	32.06917	28.08333
11		11	1	32.06861	28.08333
12		12	1	32.06806	28.08333
13		13	1	32.06667	28.08361
14		14	1	32.07833	28.08722
15		15	4	32.07194	28.07889
16		16	4	32.07138	28.07889
17		17	5	32.07083	28.07889
18	**3**	18	1	32.03278	28.07028
19		19	2	32.03278	28.07139
20		20	1	32.03389	28.06778
21		21	1	32.03278	28.06778
22		22	2	32.03333	28.06778
23	**4**	23	1	32.11611	28.09389
24		24	1	32.10222	28.10917
25		25	2	32.10167	28.10889
26		26	1	32.10139	28.10861
27	**5**	27	1	32.09611	28.09361
28		28	1	32.09611	28.09333
29		29	6	32.09056	28.09138
30		30	5	32.09000	28.09111
31	**6**	31	1	32.03250	28.10361
32		32	1	32.03139	28.10361
33		33	1	32.03028	28.09417
34		34	1	32.02722	28.08750
35		35	2	32.02778	28.08750
36		36	1	32.02833	28.08750
37	**7**	37	1	31.96917	28.16444

Sample number	Grid number	Transect number	*N*	Latitude	Longitude
38		38	1	31.96972	28.16444
39		39	2	31.96000	28.17306
**40**		40	1	31.96806	28.16472
41		41	1	31.96750	28.16472
42		42	1	31.96139	28.17333
43		43	2	31.96194	28.17333
44	**9**	44	1	32.03528	28.19472
45		45	1	32.03472	28.19417
46		46	1	32.03389	28.19417
47		47	2	32.03194	28.19611
48		48	2	32.03139	28.19611
49	**10**	49	3	31.95805	28.19778
50		50	1	31.95805	28.19833
51		51	3	31.95444	28.20389
52		52	3	31.95389	28.20389
53		53	2	31.95333	28.20389
54	**11**	54	4	31.91944	28.19417
55		55	4	31.92000	28.19417
56		56	2	31.92028	28.19417
57	**12**	57	4	31.89111	28.19278
58		58	7	31.89111	28.19333
59		59	4	31.89111	28.19389
60	**13**	60	1	31.82806	28.24694
61		61	1	31.82917	28.23194
62		62	1	31.82972	28.23194
63		63	1	31.83028	28.23194
64		64	6	31.81472	28.23722
65		65	3	31.81417	28.23722
66	**14**	66	2	31.79250	28.23944
67		67	1	31.79194	28.23944
68		68	1	31.79139	28.23944
69	**15**	69	1	31.73333	28.25667
70		70	2	31.73333	28.25778
71		71	3	31.73694	28.23056
72		72	5	31.73750	28.23056
73		73	2	31.73806	28.23056
74	**16**	74	9	31.96861	28.27583
75		75	7	31.96917	28.27583
76	**17**	76	2	31.94000	28.28306
77		77	1	31.94417	28.27667
78		78	1	31.94389	28.27694
79		79	1	31.94444	28.27639
80		80	1	31.94528	28.27667
81		81	1	31.94528	28.27722
82	**18**	82	1	31.87833	28.30361
83	**21**	83	2	31.73111	28.30944

Sample number	Grid number	Transect number	*N*	Latitude	Longitude
84		84	1	31.73222	28.31250
85	**22**	85	3	31.95917	28.40556
86		86	3	31.95917	28.40528
87		87	1	31.95861	28.40528
88		88	2	31.95778	28.40611
89		89	1	31.95778	28.40556
90	**23**	90	1	31.91444	28.41389
91		91	3	31.89944	28.40833
92		92	4	31.93222	28.40833
93	**24**	93	1	31.85083	28.42250
94		94	1	31.85056	28.42250
95	**25**	95	1	31.81944	28.42970
96		96	1	31.82694	28.42528
97		97	1	31.84056	28.41806
98		98	1	31.84000	28.41806
99	**26**	99	4	31.77639	28.42389
100		100	7	31.77639	28.42333
101		101	4	31.77639	28.42278

Table S2 Permits and permit numbers.

COUNTRY	ORGANISATION	TYPE	NUMBER	HOLDER
United Kingdom	DEFRA	Import	POAO/2011/145	I.M. Russo
South Africa	Ezemvelo KZN Wildlife	Collection	OP 614/2011	I.M. Russo
South Africa	Ezemvelo KZN Wildlife	Export	OP 1056/2011	I.M. Russo

Table S3 Matrix of relative support (RS) of the model in the column as compared to the model in the row. Model abbreviations refer to Table 3. The best-supported model is indicated in bold.

Model	***A + L***	***A + TC***	***IBR***
A + L	-	**0.042 (0.002)**	-0.018 (0.001)
A + TC	**-0.042 (0.077)**	**-**	**-0.110 (0.472)**
IBR	0.018 (0.023)	**0.110 (0.027)**	-

Methods

Landscape resistance hypotheses

Raster maps at 50 m resolution were provided by Ezemvelo KZN Wildlife, Hluhluwe Research Centre, Geographic Information Systems Unit. We analysed a comprehensive suite of landscape features (slope aspect, rivers, roads, topographic complexity and land cover; see Supplementary Fig. S1 online), based on both published and expert knowledge on the ecology and behaviour of *M. natalensis*. Hypotheses were tested according to mathematical equations (see Supplementary Equation S1 and S2) with the following parameters: x (contrast) and R_{max} (magnitude of the relationship). We varied parameter values in order to identify the most biologically accurate relationship between landscape resistance and genetic distance. Once the best parameter values have been identified, a series of partial Mantel tests in a reciprocal causal modelling framework based on RS/reduced models and mixed effect models were used to evaluate the landscape variables.

Landscape resistance was modelled as a function of aspect according to the hypothesis that there is an optimal aspect (less resistance) associated with the availability of water and favourable vegetation. McCune and Keon[1] proposed an index of heat load (Heat load index = |180 - |Aspect – 315||) such that southeast aspects (135°) represent the coolest slope and northwest aspects (315°) represent the warmest slope to create a continuous variable from 0 (cooler slopes) to 1 (warmer slopes)[2]. To test this, a modified heat load index equation, to take the southern hemisphere into account was applied (adapted from Castillo *et al.*[3]). Five maximum resistance values (R_{max}) were tested (2, 10, 100, 500 and 1 000) with five values of x (0.5, 1, 2, 4, and 10). We reclassified the aspect raster map so that flat areas had a value of $R_{max}/2$. A total of 200

candidate resistance models were created for slope aspect by testing θ_{opt} in 45° increments from 0° to 315° (see Supplementary Equation S1 & Fig. S2 online).

We hypothesised that both rivers and roads act as physical barriers to small mammal movement and are therefore negatively correlated with gene flow. Resistance was modelled as a function of water bodies including the four major rivers and the smaller rivers/streams. Landscape resistance maps were classified with a water-land dichotomy where water was assigned an $R_{\max}$ value of 2, 5, 10, 50, 100, 250, 500, 750, 1 000, 5 000 or 10 000 and land a value of 1. We also classified rivers using two categories: major rivers vs. smaller rivers/streams. Smaller rivers/streams received a resistance value of $R_{\max}/2$ except in the case where $R_{\max} = 2$ where smaller rivers/streams were assigned a value of 1.5. Likewise, we used the same two classification schemes for roads.

Topographic complexity (TC) was modelled under the hypothesis that resistance to gene flow increases as a landscape becomes more complex because of greater energetic cost to movement. Here, we reclassified the DEM using the Surface Relief Ratio (SRR) tool in ArcGIS v 10.1[4] using six radii as the number of neighbouring cells (1, 2, 5, 10, 25, 50). A power function was used with the same values for $R_{\max}$ and x as for aspect (see Supplementary Equation S2 & Fig S3 online).

Finally, we modelled resistance as a function of land cover (vegetation type) by using four classification schemes. Land cover that provides a source of food and cover/protection against predators was hypothesised to promote gene flow. An array of six resistance values (1, 168, 334, 501, 667, 1 000) was populated using 1 and a 1 000 as the minimum ($R_{\min}$) and maximum ($R_{\max}$) respectively with the four intermediate values chosen at random following a linear distribution. Without rearranging the sixth

value (1 000) that was assigned to water bodies, all 120 permutations of the remaining elements in the array were tested. Secondly, based on the species' affinity for seasonally available water we tested the dichotomy that favourable/closed habitat (R_{FH}; thicket) poses less resistance to movement that non-favourable/open habitat (all other land cover types). Landscape resistance maps were also classified with a closed-open habitat dichotomy where minimum resistance (R_{min}) was assigned to closed habitat (thicket, grassland and woodland) and open habitat received maximum resistance (R_{max}). A resistance value of 1 was assigned to closed habitat and 12 values of R_{max} were tested for open habitat (2, 5, 10, 50, 100, 250, 500, 750, 1 000, 5 000, 10 000, 100 000). Lastly, we reclassified the land cover map into six categories based on the amount of cover provided: thicket, woodland, grassland, forest, river forest and major rivers. Each cover class was ranked as a categorical variable according to the hypothesised relative resistance provided: the lowest rank followed by all the other cover types and water as a barrier with the highest resistance. These rasters were reclassified according to the function $R = \text{Rank}^x$ as described in Castillo *et al.*[3] with $R_{FH} = R_1 = 1$ and $R_6 = R_{max}$.

References

1. McCune, B. & Keon, D. Equations for potential annual direct incident radiation and heat load. *J. Veg. Sci.* **13,** 603-606 (2002).
2. McCune, B. Improved estimates of incident radiation and heat load using non-parametric regression against topographic variables. *J. Veg. Sci.* **18,** 751-754 (2007).

3. Castillo, J.A., Epps, C.W., Davis, A.R. & Cushman, S.A. Landscape effects on gene flow for a climate-sensitive montane species, the American pika. *Mol. Ecol.* **23,** 843-856 (2014).
4. Evans, J.S., Oakleaf, J., Cushman, S.A. & Theobald D. An ArcGIS Toolbox for Surface Gradient and Geomorphometirc Modeling, version 2.0-0, 2014. Available: http://evansmurphy.wix.com/evansspatial (Accessed: 2 December 2014).
